# Clinicopathological impacts of high c-Met expression in head and neck squamous cell carcinoma: a meta-analysis and review

**DOI:** 10.18632/oncotarget.21303

**Published:** 2017-09-28

**Authors:** Jung Han Kim, Bum Jun Kim, Hyeong Su Kim

**Affiliations:** ^1^ Division of Hemato-Oncology, Department of Internal Medicine, Kangnam Sacred-Heart Hospital, Hallym University Medical Center, Hallym University College of Medicine, Seoul, Republic of Korea; ^2^ Department of Internal Medicine, National Army Capital Hospital, The Armed Forces Medical Command, Sungnam, Gyeonggi-do, Republic of Korea

**Keywords:** c-Met, head and neck squamous cell carcinoma, prognosis, meta-analysis, review

## Abstract

High c-Met expression has been observed in head and neck squamous cell carcinoma (HNSCC). However, its clinicopathological impact remains controversial. We performed this meta-analysis to evaluate the pathologic and prognostic impacts of c-Met overexpression in patients with HNSCC. A systematic computerized search of the electronic databases was carried out. From 16 studies, 1,948 patients with HNSCC were included in the meta-analysis. Compared with HNSCCs showing low c-Met expression, tumors with high c-Met expression were significantly associated with higher rate of lymph node metastasis (odds ratio = 3.26, 95% CI: 2.27–4.69, *P* < 0.00001) and higher T stage (odds ratio = 1.33, 95% CI: 1.03–1.71, *P* = 0.03). In addition, patients with c-Met-high HNSCC showed significantly worse disease-free survival (hazard ratio = 1.49, 95% CI: 1.04–2.14, *P* = 0.03) and overall survival (hazard ratio = 1.83, 95% CI: 1.29–2.60, *P* = 0.0007) than those with c-Met-low tumor. In conclusion, this meta-analysis demonstrates that high c-Met expression is significantly associated with worse pathological features and prognosis, indicating c-Met overexpression is an adverse prognostic marker for patients with HNSCC.

## INTRODUCTION

Head and neck cancers (HNCs) are classified as epithelial neoplasms of the oral cavity (including tongue and tonsils), nasal cavity, paranasal sinuses, pharynx, and larynx. HNCs have been increasing worldwide, comprising one of the most common groups of cancer [1–3]. Despite the heterogeneity both in tumor location and genetic aberrations, histologically 90% of HNCs are squamous cell carcinoma (HNSCC). About two-thirds of patients with HNSCC are presented with advanced diseases at the time of diagnosis. Treatments of patients with advanced HNSCC usually involve the multitude of therapeutic modalities such as surgical resection, radiation, or concurrent chemoradiation. Although initial treatments are generally intended to give the chance to cure for patients with advanced HNSCC, however, these tumors are characterized by frequent recurrence or metastasis as well as resistance to the conventional chemoradiotherapy. Moreover, patients with recurrent or metastatic HNSCC have shown dismal outcomes [4].

With understanding of molecular mechanisms of carcinogenesis, treatment of recurrent or metastatic HNSCC has changed over the last decade. Cetuximab, a monoclonal antibody to the epidermal growth factor receptor (EGFR), was the first to receive FDA approval with survival advantage when combined with radiation or platinum-based chemotherapy [5, 6]. In 2016, pembrolizumab and nivolumab, immune checkpoint inhibitors, also received FDA approval for patients with recurrent or metastatic HNSCC. These monoclonal antibodies have shown a survival benefit when compared with standard care of chemotherapies [7, 8]. However, most tumors develop resistance to the molecular targeted agents and their survival advantages are still disappointing. Therefore, there is still a need to identify novel therapeutic targets promoting HNSCC pathogenesis and develop more efficacious targeted agents. The c-Met/hepatocyte growth factor (HGF) pathway has recently emerged as a potential therapeutic target in various tumors including HNSCC [9, 10].

c-Met, the tyrosine kinase receptor for HGF, is encoded by the proto-oncogene *MET* located on chromosome 7 [11]. The dysregulation of the HGF/c-Met signaling pathway has been implicated in the pathogenesis of cancer, such as tumor cell proliferation and survival, invasion, and metastasis [12, 13]. In addition to amplification, mutation, or transcriptional alteration of *MET*, c-Met may be activated by protein overexpression or paracrine/autocrine signaling of HGF [14–16]. The overexpression of c-Met has been observed in various types of tumors, such as hepatocellular carcinoma [16], breast cancer [17], lung cancer [18], gastric cancer [19], colorectal cancer [20], cervical cancer [21], renal cell carcinoma [22], and pancreatic cancer [23].

The expression of c-Met has also been detected in HNSCC [24–45]. Many studies have reported that high c-Met expression is significantly associated with poor pathologic features and/or prognosis in HNSCC. Because of the small number of patients and variability of detection methods in most studies, however, there have been some conflicts regarding its pathologic or prognostic impact [36, 39, 40]. We conducted this meta-analysis to evaluate the clinicopathological roles of high c-Met expression in patients with HNSCC.

## RESULTS

### Results of search

**Figure 1:**
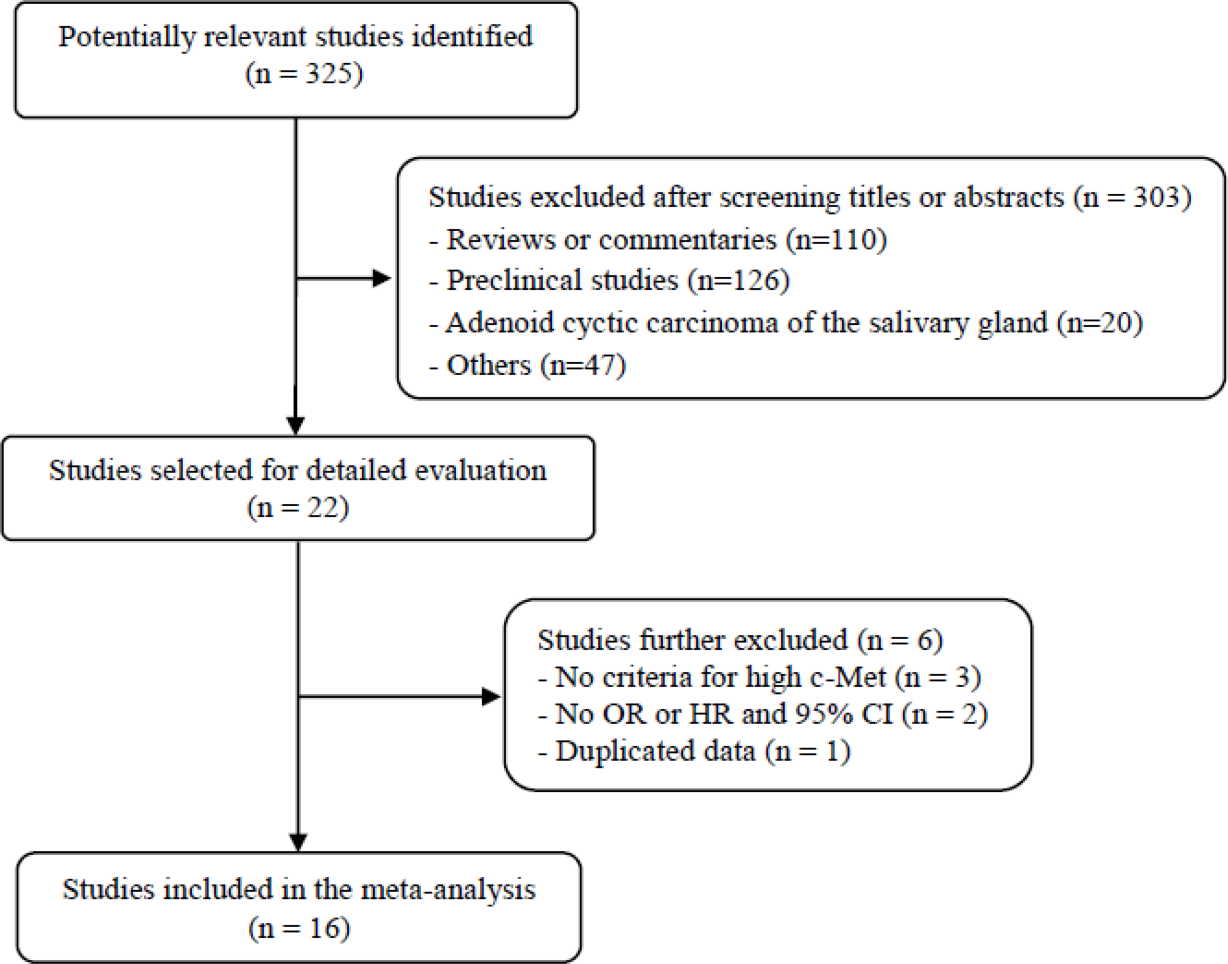
Flow diagram of search process

Figure 1 shows flow diagram of search process. A total of 325 potentially relevant studies were initially found, but 303 of them were excluded after screening the titles and abstracts. Of the remaining 22 potentially eligible studies, 6 were further excluded by the inclusion criteria: three had no definite criteria for high c-Met expression [24–26]; two had no available data to estimate hazard ratio (HR) or odds ratio (OR) with 95% confidence interval (CI) [27, 28]; one included duplicated data [29]. Finally, sixteen studies were included in the meta-analysis [30–45].

### Characteristics of the included studies

Supplementary Table 1 summarizes the main characteristics and clinicopathological findings of the included studies. Most studies were performed retrospectively. From the 16 studies, 1,948 patients were included in the meta-analysis. Except for one with recurrent or metastatic HNSCCs [43], most studies had patients with a locoregionally advanced disease.

In 13 studies [30–40, 44, 45], patients underwent surgical resection without neoadjuvant therapy. In two studies [41, 42], patients were treated with concurrent chemoradiation or radiation alone as a first-line treatment. All the studies used immunohistochemistry (IHC) to assess c-Met expression status but adopted various cutoff values for high c-Met expression.

### c-Met expression assignation

There was a marked heterogeneity in the criteria used to dichotomize c-Met expression status (low or high) among studies. The IHC criteria were briefly summarized in the Supplementary Table 1. The rates of high c-Met expression were various, ranging from 26% [31] to 82.9% [36].

### Impact of high c-Met expression on pathological features

**Figure 2:**
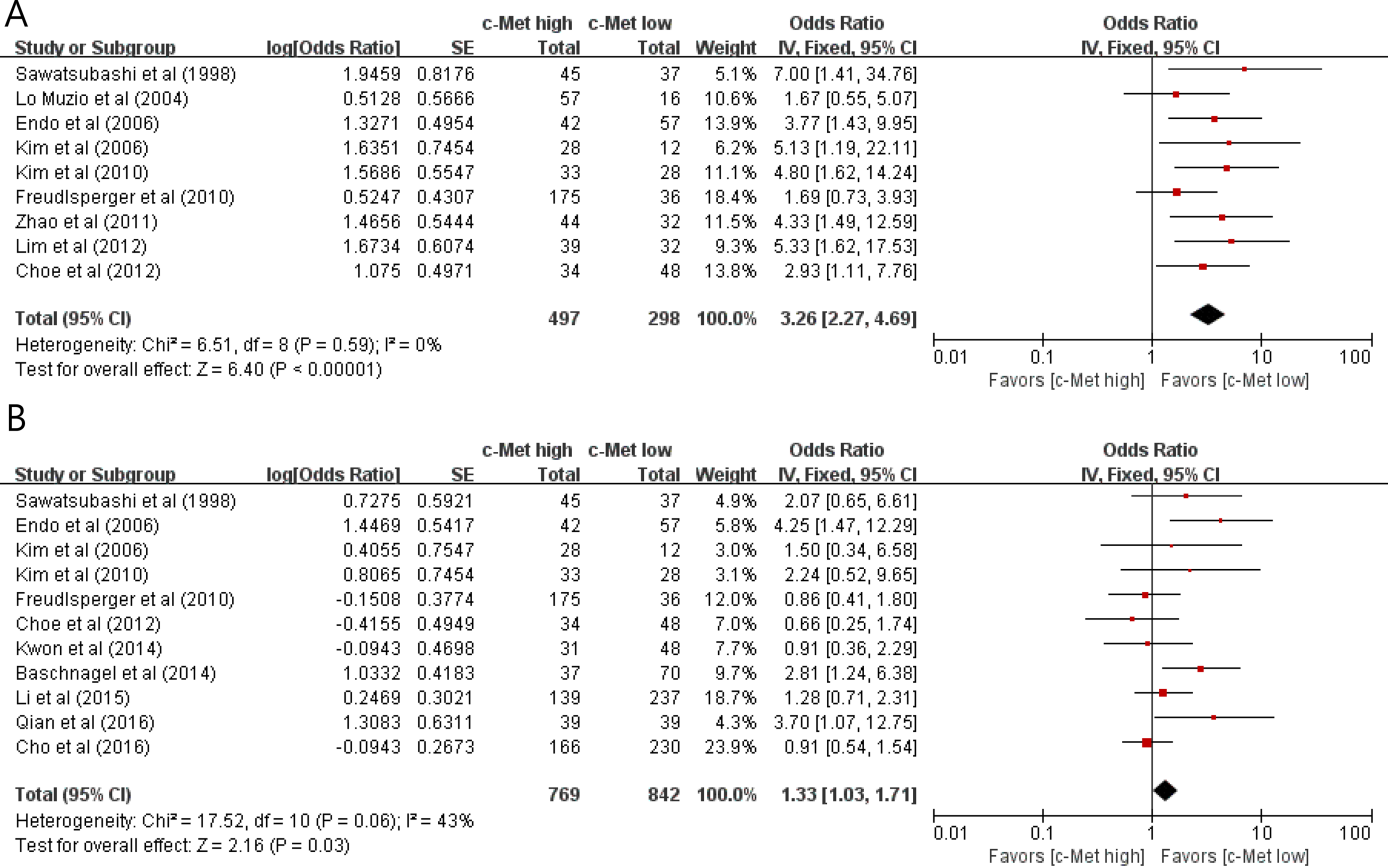
Forest plots of odds ratios for lymph node metastasis (A) and T stage (B)

From nine studies [30–33, 35–39], 795 patients were included in the meta-analysis of odds ratios (ORs) with 95% confidence intervals (CIs) for lymph node (LN) metastasis. Compared with HNSCCs with low c-Met expression, tumors with high c-Met expression showed significantly higher rate of LN metastasis (OR = 3.26 [95% CI, 2.27–4.69], *P* < 0.00001) (Figure 2A). The fixed-effects model was selected because there was no significant heterogeneity among studies (*X*^2^ = 6.51, *P* = 0.59, *I^2^* = 0%).

From eleven studies [30, 32, 33, 35, 36, 39–42, 44], 1,611 patients were included in the meta-analysis of ORs with 95% CIs for tumor *T* stage. There was a positive correlation between c-Met overexpression and higher T classification (T3 and T4) (OR = 1.33 [95% CI, 1.03–1.71], *P*= 0.03) (Figure 2B). The fixed-effects model was used because there was little heterogeneity among studies (*X*^2^ = 17.52, *P* = 0.06, *I^2^* = 43%).

### Impact of high c-Met expression on survival

**Figure 3:**
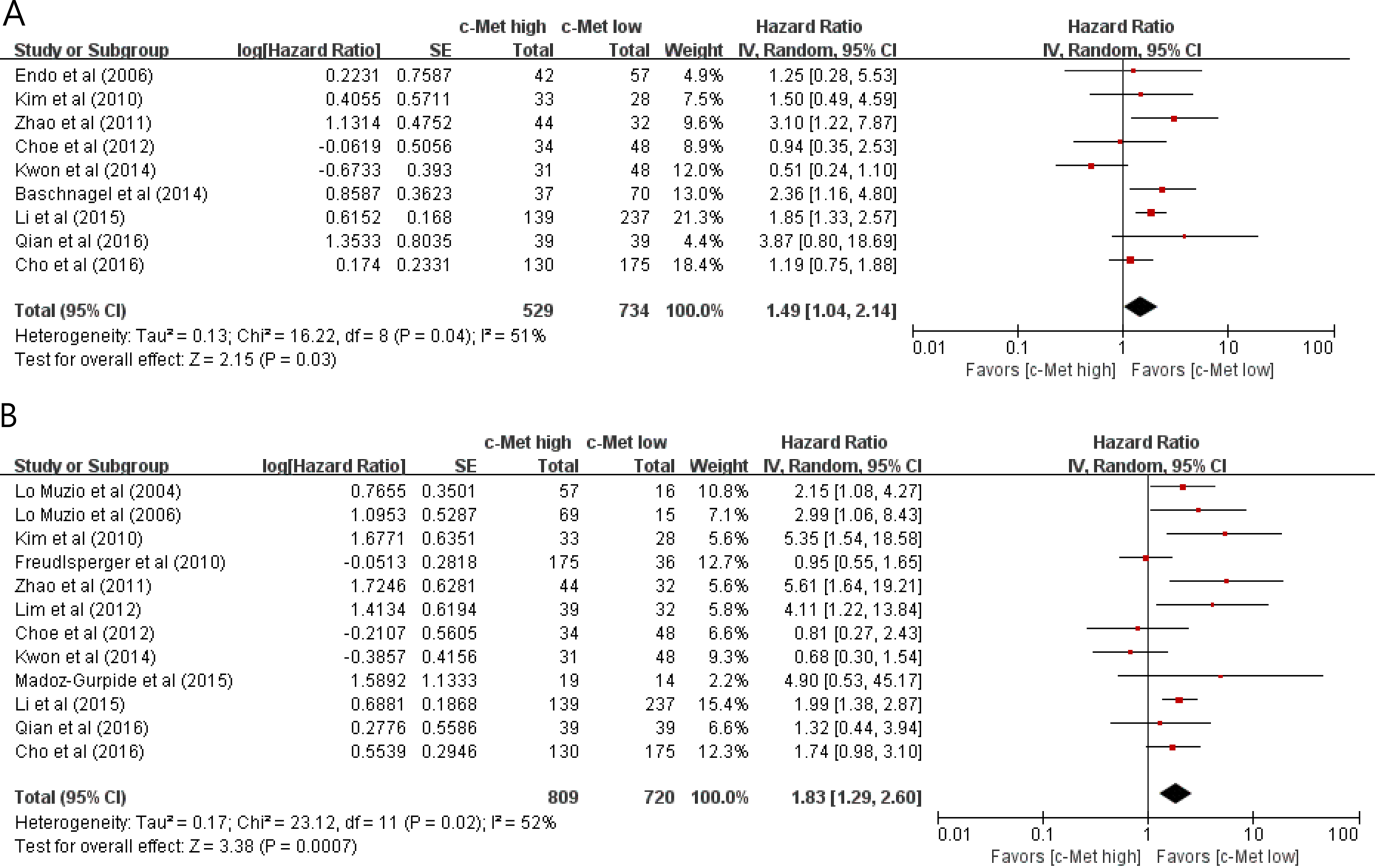
Forest plots of hazard ratios for disease-free survival (A) and overall survival (B)

From 9 studies [32, 35, 37, 39–42, 44, 45], 1,263 patients were included in the meta-analysis of HRs with 95% CIs for disease-free survival (DFS). Patients with c-Met-high HNSCC showed significantly worse DFS than those with c-Met-low tumor (HR = 1.49 [95% CI, 1.04–2.14], *P* = 0.03) (Figure 3A). The random-effects model was selected because there was a significant heterogeneity across the studies (*X*^2^ = 16.22, *P* = 0.04, *I^2^* = 51%).

From 12 studies [31, 34–40, 42–45], 1,529 patients were included in the meta-analysis of HRs with 95% CIs for overall survival (OS). Patients with c-Met-high HNSCC showed significantly poor OS (HR = 1.83 [95% CI, 1.29–2.60], *P* = 0.0007) (Figure 3B), compared with those with c-Met-low tumor. The random-effects model was used because there was a significant heterogeneity across the studies (*X*^2^ = 23.12, *P* = 0.02, *I^2^* = 52%).

### Publication bias

**Figure 4:**
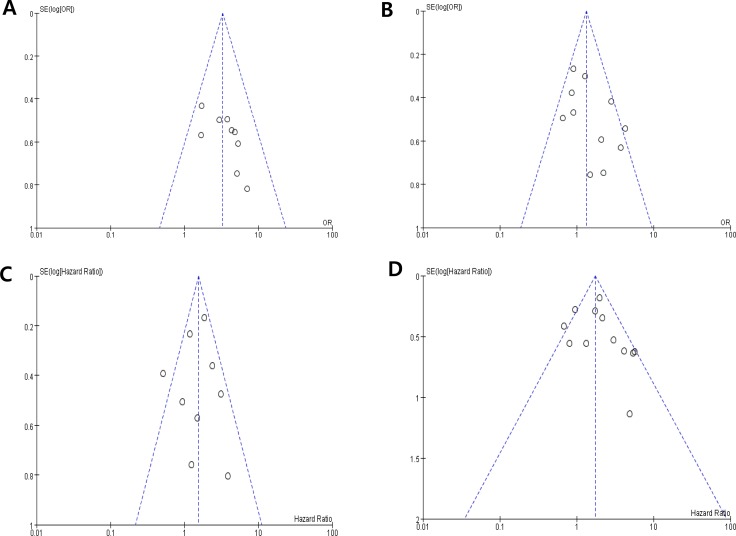
Funnel plots for publication bias regarding lymph node metastasis (A), T stage (B), disease-free survival (C) and overall survival (D)

Visual inspection of the funnel plots for LN metastasis, T stage, DFS and OS showed symmetry, indicating there were no substantial publication biases (Figure 4A–4D).

## DISCUSSION

In this meta-analysis, we investigated the pathologic and prognostic impact of high c-Met expression in patients with HNSCC. The results show that c-Met overexpression significantly correlated with poor pathological features and prognosis. To our knowledge, this is the first meta-analysis to evaluate the clinicopathological impacts of high c-Met expression in patients with HNSCC.

*MET* activation has been proven to play a critical role in the pathogenesis and progression of many tumor types [14–16]. Mechanisms of *MET* activation include mutations, amplification, and overexpression of both c-Met and HGF protein [15, 16]. *MET* amplification has been detected in up to 13% of tumors and *MET* mutations are less common in HNSCCs [46, 47]. *MET* amplification has been proposed as an independent prognostic factor for DFS and OS in locoregionally advanced HNSCC [42]. While *MET* amplification and increased *MET* gene copy number have been detected at a low frequency in HNSCC tumors, they are associated with the overexpression of c-Met protein [43, 45]. c-Met overexpression is the most frequently observed alteration presenting in up to 80% in HNSCCs [34, 36]. Many studies in HNSCC have suggested that c-Met expression is correlated with clinicopathological parameters indicative of poor prognosis, such as differentiation [39], T classification [25, 41, 44], LN metastasis [25, 30, 33, 35, 37–39], disease stage [25, 32, 35], and worse DFS [24, 37, 41, 42] or OS [24, 31, 34, 35, 37, 38, 42]. However, the pathological or clinical impacts of c-Met expression were not consistent across the studies [26–28, 32, 36, 39, 40, 45]. For example, Freudlsperger *et al*. evaluated c-Met expression in 211 patients with oral cavity SCC and failed to find any prognostic impact in regard to tumor size or stage, LN metastasis, and OS [36]. Potential explanations for these discrepancies may stem from the heterogeneity of patients and diverse primary tumor sites. In addition, most studies had a small number of patients and adopted different cut-off values for high versus low c-Met expression levels.

In the current meta-analysis, we included studies comparing the major pathological features (LN metastasis and T classification) and survival outcomes (DFS and OS) according to the c-Met expression status. All the studies used IHC to test c-Met expression. Compared with HNSCCs showing low c-Met expression, tumors with high expression showed significantly higher rate of LN metastasis (OR = 3.26, *P* < 0.00001) and higher T-stage (OR = 1.33, *P* = 0.03). In addition, patients with c-Met-high HNSCC showed significantly worse DFS (HR = 1.49, *P* = 0.03) and OS (HR = 1.83, *P* = 0.0007) than those with c-Met-low tumor. Our findings indicate that high c-Met expression represent a significant adverse prognostic marker in patients with HNSCC.

Several meta-analyses in other cancers have also defined high c-Met expression as an adverse prognostic marker for survival [17–21]. Therefore, inhibition of c-Met/HGF signaling may provide an effective therapeutic strategy for cancers showing high c-Met expression [9, 10, 48]. With ample evidence for the role of the c-Met/HGF pathway promoting tumor progression, various c-Met inhibitors are under active investigation in a variety of cancers, including HNSCC [48–52]. Seiwert *et al.* conducted the first phase II trial to evaluate a c-Met inhibitor in HNSCCs [49]. They tested the efficacy and safety of single agent foretinib (a multi-kinase inhibitor targeting c-Met, VEGF2, RON, AXL, and TIE-2 receptors) in patients with recurrent or metastatic HNSCC. Although no patients achieved objective response, half of the patients (7/14) showed stable disease, with minor tumor shrinkage in 6. The predictive role of c-Met expression could not be evaluated due to the small sample size, but the results supported the further investigation of c-Met inhibitors for HNSCC. Interestingly, the efficacy of c-Met-targeting agents has been associated with high c-Met expression in other tumors including non-small-cell lung cancer, hepatocellular carcinoma, and renal cell carcinoma [50–52]. These results suggest that patients with cancer showing high c-Met expression may be good candidates for c-Met inhibitors.

Human papillomavirus (HPV) and EGFR are biomarkers that have been extensively studied in HNSCCs. Some studies have suggested that aberrant c-Met/HGF signaling is associated with HPV status [41, 44]. Qian *et al.* reported that high c-Met expression was associated with HPV-positive status in patients with oropharyngeal SCC [44]. In the study of patients with locally advanced HNSCC treated with chemoradiation by Baschnagel *et al.*, high c-Met expression predicted for worse DFS in p16-negative patients but not in those with p16-positive tumor [41]. However, there was no significant correlation between c-Met expression and HPV status in other studies [39, 40, 45]. EGFR is highly overexpressed and correlates with disease progression in HNSCC [53]. In the study by Baschnagel *et al.* high c-Met expression was associated with EGFR positivity [41]. However, c-Met overexpression was prognostic in both EGFR-positive and EGFR-negative patients. Unfortunately, we could not include HPV status and EGFR status in this meta-analysis because of limited data available.

Recently, c-Met activation has been proposed as a potential mode of resistance to anti-EGFR therapy in HNSCC [54–57]. The c-Met/HGF signaling pathway has cross-talks with the EGFR network at both PI3K/Akt and MAPK nodes, suggesting mutual compensation. c-Met has been observed to be coexpressed with EGFR in HNSCC cell lines [56] and it has been identified as a marker of cisplatin and erlotinib resistance [54, 57]. In a retrospective study of recurrent or metastatic HNSCCs treated with cetuximab, patients with c-Met overexpression showed a significantly worse progression-free survival (HR = 7.6 [95% CI, 4.6–10.4], *P* = 0.06) and OS (HR = 4.9 [955 CI, 0.1–8.5], *P* = 0.07) [43]. These findings indicate that c-Met expression may serve as a biomarker to predict who benefit less from anti-EGFR therapy. In preclinical models of HNSCC, in addition, knockdown of c-Met has enhanced sensitivity of cancer cells to anti-EGFR agents [54, 55]. These results suggest that c-Met inhibitors may overcome resistance to anti-EGFR therapy in recurrent or metastatic HNSCC.

The major challenge for clinical development of c-Met inhibitors is that there is no consensus of the reliable criteria for c-Met overexpression. A variety of methods, such as IHC, Western blot, fluorescence *in situ* hybridization, and real-time quantitative PCR are currently used to test c-Met expression, but there are no standardized criteria for overexpression. The discrepancies in the clinicopathological impacts of c-Met among studies might be attributable to the different methods and criteria for high c-Met expression. Therefore, the definition of reliable criteria for c-Met status is essential to verify the prognostic role of c-Met expression and investigate the efficacy of c-Met inhibitors.

Our study has several inherent limitations. First, the included studies had various primary sites in the head and neck. Second, because of the limited number of studies, we could not perform subgroup analyses according to the primary sites and HPV status. Third, most studies were retrospectively carried out. Fourth, although almost all patients had SCC of the head and neck, some studies included patients with undifferenciated carcinoma; however, because they occupied only a very small portion of patients, inclusion of these patients does not seem to affect the results. Fifth, while most studies had patients with a locoregionally advanced disease, one was conducted in recurrent or metastatic setting [43]. Because this study met the inclusion criteria, providing the survival data according to c-Met status, we included it in the meta-analysis of OS. Finally, as we mentioned above, the studies used different IHC methods to test c-Met expression and adopted various cut-off values to stratify c-Met status.

In conclusion, this meta-analysis demonstrates that c-Met overexpression is significantly associated with poor pathological features and prognosis. These findings indicate that high c-Met expression is a potential adverse prognostic marker for patients with HNSCC. However, larger studies using standardized methods and criteria are still needed to verify the prognostic role of c-Met expression in HNSCC with various primary sites.

## MATERIALS AND METHODS

### Search strategy

This study was performed according to the Preferred Reporting Items for Systematic Reviews and Meta-Analyses (PRISMA) guidelines [58]. We carried out a computerized electronic search of the databases such as PubMed, Embase, and Google Scholar (up to May 2017). The search used the following keyword: “c-Met” or “Met”, “hepatocyte growth factor receptor”, and “head and neck cancer” or “head and neck squamous cell carcinoma”. The related articles function of the PubMed was also used to identify all relevant articles. The titles and abstracts of retrieved studies were carefully scanned to exclude irrelevant papers. Then, the potentially eligible articles were reviewed in full text and those that did not meet the selection criteria were further excluded. For the potential duplicate articles, only the most complete study was included.

### Inclusion criteria

Eligible studies were required to meet the following inclusion criteria: (i) patients had a pathological diagnosis of head and neck squamous cell carcinoma; (ii) articles had criteria for high c-Met expression; (iii) pathological features (LN metastasis or T classification) and/or survival outcomes (DFS or OS) were stratified according to c-Met expression status; (iv) sufficient data were provided to estimate OR or HR with 95% CI; (v) articles were published in English.

### Data extraction

The data were collected independently by two investigators (BJK and HSK). If these two authors did not agree, the other investigator (JHK) was consulted to resolve the discrepancies.

The following data were extracted from all eligible studies: the first author's name, publication year, country, number of patients, tumor sites, T classification, LN metastasis, primary treatment, methods to test c-Met expression, cut-off values adopted to dichotomize c-Met expression status, and HR with 95% CI for DFS or OS and OR with 95% CI for pathological features.

### Statistical analysis

Statistical values were obtained directly from the original articles. If OR or HR with 95% CI were not provided, the Engauge Digitizer (version 9.1) was used to estimate the needed data from the results and Kaplan-Meier curves. The strength of the association between c-Met overexpression and pathologic features (LN metastasis or T stage) was shown as ORs with their 95% CIs. The effect size of DFS and OS was pooled through HR with its 95% CI. The heterogeneity across studies was tested by the *Q* statistic and the *I*^2^ inconsistency test. The fixed-effects model (Mantel–Haenszel method) was selected for pooling homogeneous outcomes when *P*≥ 0.1 and *I^2^ ≤* 50%, whereas the random-effects model (DerSimonian–Laird method) was applied when there was a significant heterogeneity (*P* < 0.1 and *I^2^* > 50%). The RevMan software (version 5.2) was used to combine data and report outcomes. All *P*-values were two-sided and *P* < 0.05 was considered statistically significant. Publication bias was assessed graphically by the funnel plot method [59].

## SUPPLEMENTARY MATERIALS TABLE




