# Supplementary material for: Clinicopathological impacts of high c-Met expression in head and neck squamous cell carcinoma: a meta-analysis and review
Source: Oncotarget. 2017 Sep 28;8(68):113120–8. doi: 10.18632/oncotarget.21303 (PMC5762576; doi:10.18632/oncotarget.21303)
Supplement: Supplementary file 1 [file oncotarget-08-113120-s001.pdf]

Clinicopathological impacts of high c-Met expression in head and neck squamous cell carcinoma: a meta-analysis and review

SUPPLEMENTARY MATERIALS

Supplementary Table 1: Summary of the 16 included studies. See Supplementary_Table_1
