# Supplementary material for: Clinicopathological impacts of high c-Met expression in head and neck squamous cell carcinoma: a meta-analysis and review
Source: Oncotarget. 2017 Sep 28;8(68):113120–8. doi: 10.18632/oncotarget.21303 (PMC5762576; doi:10.18632/oncotarget.21303)
Supplement: Supplementary file 2 [file oncotarget-08-113120-s002.docx]

**Supplementary Table 1.** Summary of the 16 included studies

| First author  (year) [ref]  Country | No. of pts | Sites | Primary  treatment | Materials,  Antibody | IHC criteria for c-Met expression | High c-Met  n (%) | OR for LN mets  (95% CI) | OR for T3/4  (95% CI) | HR for DFS  (95% CI) | HR for OS  (95% CI) |
| --- | --- | --- | --- | --- | --- | --- | --- | --- | --- | --- |
| Sawatsubashi  (1998) [30]  Japan | 82 | Larynx | Surgery or unknown | Whole slides,  Polyclonal anti-c-Met (C-12) | 0 (no staining); +/- (definite, but weak); + (moderate); ++ (strong)  (high c-Met: scored + or ++) | 45 (54.9%) | 7.00 (1.41-34.76)  *P* = 0.032 | 2.07 (0.65-6.61)  NS | NA | NA |
| Lo Muzio  (2004) [31]  Italy | 73 | Oral cavity | Surgery | Whole slides,  Polyclonal anti-c-Met (C-12) | 0 (no or weak staining in < 10% of tumor cells); 1 (weak to moderate in 10-30%); 2 (moderate in 31-50%); 3 (strong in ≥ 50%)  (high c-Met: score 3) | 19 (26%) | 1.67 (0.55-5.07)  NS | NA | NA | 2.15 (1.08-4.27)  *P* = 0.0342 |
| Endo  (2006) [32]  Japan | 99 | Tongue | Surgery | Whole slides,  Anti-c-Met (C-28) | (high c-Met: positive cells ≥ 45% of tumor cells) | 42 (42.4%) | 3.77 (1.43-9.95) | 4.25 (1.47-12.29) | 1.245 (0.28-5.48)  *P* = 0.772 | NA |
| Kim  (2006) [33]  Korea | 40 | Hypopharynx | Surgery | Whole slides,  Human polyclonal anti-c-Met | - (no staining); +/- (definite but weak); + (moderate); 2+ (strong)  (high c-Met: scored + or 2+ and stained > 30% of cancer cells) | 28 (70%) | 5.13 (1.19-22.11)  *P* = 0.032 | 1.50 (0.34-6.58)  *P* = 0.704 |  | NA  NS |
| Lo Muzio  (2006) [34]  Italy | 84 | Oral cavity | Surgery | Whole slides,  Polyclonal anti-c-Met (C-12) | 1 (staining in 0-30% of total cancer cells); 2 (staining in ≥ 30%)  (high c-Met: score 2) | 69 (82.2%) | NA | NA | NA | 2.99 (1.06-8.43)  *P* = 0.0305 |
| Kim,  (2010) [35]  Korea | 61 | Tongue | Surgery | Whole slides,  Human polyclonal anti-c-Met (C-28) | 0 (no staining); +/- (definite but weak); + (moderate); ++ (strong)  (high c-Met: score + or ++ and stained ≥ 30% of tumor cells) | 33 (54.1%) | 4.80 (1.62-14.24)  *P* = 0.005 | 2.24 (0.52-9.65)  *P* = 0.319 | 1.5 (0.50 – 4.69)  *P* = 0.452 | 5.35 (1.54-18.58)  *P* = 0.003 |
| Freudlsperger  (2010) [36]  Germany | 211 | Oral cavity | Surgery | Whole slides,  Anti-c-Met  (1:50) | (high c-Met: ≥ 50% staining of cancer cells) | 175(82.9%) | 1.69 (0.73-3.93)  *P* = 0.242 | 0.86 (0.41-1.80)  *P* = 0.706 | NA | 0.95 (0.55-1.65)  *P* = 0.8244 |
| Zhao  (2011) [37]  China | 76 | Oral cavity | Surgery | Whole slides,  Anti-c-Met | 0 (no staining); +/- (definite but weak); + (moderate); ++ (strong)  (high c-Met: scored + or ++) | 44 (57.9%) | 4.33 (1.49-12.59)  *P* = 0.005 | NA | 3.10 (1.22-7.87)  *P* = 0.010 | 5.61 (1.64-19.21)  *P* < 0.00 |
| Lim  (2012) [38]  Korea | 71 | Tongue | Surgery | Whole slides,  Human polyclonal anti-c-Met | 0 (no staining); +/- (definite but weak); + (moderate); ++ (strong)  (high c-Met: scored + or ++ and stained ≥ 30% of cancer cells)) | 39 (54.9%) | 5.33 (1.62-17.52)  *P* = 0.006 | NA |  | 4.11 (1.22-13.84)  *P* = 0.018 |
| Choe  (2012) [39]  Korea | 82 | Larynx (29)  Nasopharynx (19)  Oropharynx (17)  Oral cavity (13)  Hypopharynx (3) | Surgery | TMA,  Rabbit anti-c-Met | Intensity: graded from 0 to 3  Frequency of positivity portion: 0 (< 1%); 0.1 (1-9%); 0.5 (10-49%); 1 (50-100%)  (high c-Met: intensity x frequency > 1) | 34 (41.5%) | 2.93 (1.11-7.76)  P = 0.035 | 0.66 (0.25-1.74)  P = 0.273 | 0.94 (0.35-2.53)  *P* = 0.857 | 0.81 (0.27-2.43)  *P* = 0.551 |
| Kwon  (2014) [40]  Korea | 79 | Tonsil | Surgery | TMA,  Rabbit anti-c-Met | Intensity: 0 (none); 1 (weak); 2 (moderate); 3 (strong)  Proportion: 0 (none); 1 (0-10%); 2 (11-30%); 3 (31-75%; 4 ( > 75%)  (high c-Met: intensity + proportion score ≥ 4) | 31 (39.2%) | NA | 0.91 (0.36-2.29)  *P* = 0.835 | 0.51 (0.24-1.10)  *P* = 0.096 | 0.68 (0.30-1.54)  *P* = 0.344 |
| Baschnagel  (2014) [41]  USA | 107 | Oropharynx (70)  Larynx (27)  Hypopharynx (7)  Oral cavity (3) | CRT | TMA,  c-Met clone 8F11 | 0 (no staining); 1 (weak); 2 (intermediate); 3 (intense)  (high c-Met: score 3) | 37 (34.6%) | NA | 2.81 (1.24-6.38)  *P* = 0.015 | 2.36 (1.16-4.80)  *P* = 0.018 | NA |
| Li  (2015) [42]  China | 376 | Nasopharyx | CRT or RT | Whole slides,  Rabbit monoclonal anti-c-Met (SP44) | 0 (no or < 50% staining of tumor cells); 1+ (≥ 50% with > weak intensity and < 50% with > moderate); 2+ (≥ 50% with > moderate and < 50% with strong intensity); 3+ (≥ 50% with strong intensity)  (high c-Met: scored 2+ or 3+) | 139 (37%) | NA | 1.28 (0.71-2.31)  P = 0.409 | 1.85 (1.33-2.57)  *P* < 0.001 | 1.99 (1.38-2.87)  *P* < 0.001 |
| Madoz-Gurpide  (2015) [43]  Spain | 33 | Oral cavity (7),  Oropharyx (7),  Hypopharynx (6),  Larynx (12),  NA (1) | CT | Whole slides,  Mouse monoclonal anti-c-Met (SP44) | H-score = (low %) x 1 + (medium %) x 2 + (high %) x 3  (high c-Met: cut-off point ≥ 120) | 22 (66.7%) | NA | NA | NA | 4.9 (0.1-8.5)  *P* = 0.070 |
| Qian  (2016) [44]  USA | 78 | Oropharynx | Surgery | Whole slides,  Anti-c-Met | Intensity: 0 (no staining); 1+ (weak staining); 2+ (intermediate); 3+ (strong)  Weighted index: intensity score x % of positive staining  (high c-Met: weighted index ≥ median 76.25) | 39 (50%) | NA | 3.70 1.07-12.75)  *P* = 0.039 | 3.87 (0.80-18.69)  *P* = 0.0779 | 1.32 (0.44-3.94)  *P* = 0.6785 |
| Cho  (2016) [45]  Korea | 396  (305^*^) | Oral cavity (204)  Oropharynx (122)  Larynx (42)  Hypopharynx (28) | Surgery | TMA  Anti-c-Met (SP44) | Intensity: 0 (no staining); 1 (weak or barely membranous staining); 2 (distinct brown); 3 (strong dark brown)  H-score: intensity score x % of positive cells  (high c-Met: H-score cut-off point ≥ 65) | 166 (41.9%)  130 (78.3%)^*^ | NA | 0.91 (0.54-1.54)  *P* = 0.720 | -  1.186 (0.75-1.87)^*^  *P* = 0.466 | -  1.742 (0.98-3.11)^*^  *P* = 0.061 |

CT, chemotherapy; CRT, chemoradiotherapy; TMA, tissue microarray; IHC, immunohistochemistry; pts, patients; OR, odds ratio; HR, hazard ratio; CI, confidence interval; DFS, disease-free survival; OS, overall survival; mets, metastasis; NS, not significant; NA, not available

* Patients with R0 resection
